# Surgical outcomes of gallbladder cancer: the OMEGA retrospective, multicentre, international cohort study

**DOI:** 10.1016/j.eclinm.2023.101951

**Published:** 2023-04-13

**Authors:** Anita Balakrishnan, Petros Barmpounakis, Nikolaos Demiris, Asif Jah, Harry V.M. Spiers, Shibojit Talukder, Jack L. Martin, Paul Gibbs, Simon J.F. Harper, Emmanuel L. Huguet, Vasilis Kosmoliaptsis, Siong S. Liau, Raaj K. Praseedom, Bristi Basu, Xavier de Aretxabala, Javier Lendoire, Shishir Maithel, Alejandro Branes, Bodil Andersson, Alejandro Serrablo, Volkan Adsay, The OMEGA Study Investigators, Tomoyuki Abe, Moh'd Abu Hilal, Maria del Mar Achalandabaso Boira, Mustapha Adham, Mohamed Adam, Maryam Ahmad, Bilal Al-Sarireh, Maite Albiol, Nassir Alhaboob, Adnan Alseidi, Houssem Ammar, Akshay Anand, Bodil Andersson, Pantelis Antonakis, Veronica Araya, Stanley W. Ashley, Georgi Atanasov, Fabio Ausania, Ricardo Balestri, Abhirup Banerjee, Sudeep Banerjee, Simon Banting, Giedrius Barauskas, Fabian Bartsch, Andrea Belli, Simona Beretta, Frederik Berrevoet, Ramesh Singh Bhandari, Gerardo Blanco Fernandez, Louisa Bolm, Mathieu Bonal, Emre Bozkurt, Andries E. Braat, Luke Bradshaw, Konstantinos Bramis, Alejandro Branes, Lyle Burdine, Matthew Byrne, Maria Caceres, Maria Jesus Castro Santiago, Benjamin Chan, Lynn Chong, Ahmet Çoker, Maria Conde Rodriguez, Daniel Croagh, Alyn Crutchley, Carmen Cutolo, Mathieu D'Hondt, Daniel D'Souza, Freek Daams, Raffaele Dalla Valle, José Davide, Mario de Bellis, Marieke de Boer, Celine de Meyere, Philip de Reuver, Matthew Dixon, Panagiotis Dorovinis, Gabriela Echeverría Bauer, Maria Eduarda, Hasan Eker, Joris Erdmann, Mert Erkan, Evangelos Felekouras, Emanuele Felli, Eduardo Fernandes, Eduardo Figueroa Rivera, Andras Fulop, Daniel Galun, Michael Gerhards, Poya Ghorbani, Fabio Giannone, Luis Gil, Emmanouil Giorgakis, Mario Giuffrida, Felice Giuliante, Ioannis Gkekas, Miguel Gomez Bravo, Bas Groot Koerkamp, Oscar Guevara, Alfredo Guglielmi, Aiste Gulla, Rahul Gupta, Amit Gupta, Marta Gutiérrez, Abu Bakar Hafeez Bhatti, Jeroen Hagendoorn, Zain Hajee, Abdul Rahman Hakeem, Hytham Hamid, Sayed Hassen, Stefan Heinrich, Ryota Higuchi, Daniel Hoffman, David Holroyd, Daniel Hughes, Arpad Ivanecz, Satheesh Iype, Isabel Jaen Torrejimeno, Shantanu Joglekar, Robert Jones, Klaus Kaczirek, Harsh Kanhere, Ambareen Kausar, Zhanyi Kee, Jessica Keilson, Jorg Kleef, Johannes Klose, Brett Knowles, Jun Kit Koong, Nagappan Kumar, Supreeth Kunnuru, Paleswan Joshi Lakhey, Andrea Laurenzi, Yeong Sing Lee, Felipe Leon, Voon Meng Leow, Jean-Baptiste Lequeu, Mickael Lesurtel, Elisabeth Lo, Stefan Löb, Elizabeth Lockie, Peter Lodge, Dolores López Garnica, Victor Lopez Lopez, Linda Lundgren, Nikolaos Machairas, Dhiresh Maharjan, Deep Malde, Guillaume Martel, Julie Martin, Michele Mazzola, Arianeb Mehrabi, Ricardo Memeo, Flavio Milana, George Molina, Leah Monette, Haluk Morgul, Dimitrios Moris, Antonios Morsi-Yeroyannis, Nicholas Mowbray, Francesk Mulita, Edoardo Maria Muttillo, Malith Nandasena, Pueya Rashid Nashidengo, Arash Nickkholgh, Colin Byron Noel, Masayuki Ohtsuka, Arturs Ozolins, Sanjay Pandanaboyana, Nikolaos Pararas, Alessandro Parente, June Peng, Arkaitz Perfecto Valero, Julie Perinel, Konstatinos Perivoliotis, Teresa Perra, Patrick Pessaux, Natalie Petruch, Gaetano Piccolo, Laszlo Piros, Alberto Porcu, Viswakumar Prabakaran, Raj Prasad, Mikel Prieto Calvo, Florian Primavesi, Eva Maria Pueyo Periz, Alberto Quaglia, Jose M. Ramia Angel, Ashwin Rammohan, Francesco Razionale, Ricardo Robles Campos, Manas Roy, Sophie Rozwadowski, Luis Ruffolo, Natalia Ruiz, Andrea Ruzzenante, Lily Saadat, Mohamed Amine Said, Edoardo Saladino, Gabriel Saliba, Per Sandstrom, Carlo Alberto Schena, Anthony Scholer, Christoph Schwarz, Lorenzo Serafini, Pablo E. Serrano, Deepak Sharma, Aali Sheen, Vishwanath Siddagangaiah, Michael Silva, Saurabh Singh, Ajith Siriwardena, Michal Skalski, Mante Smig, Faris Soliman, Abhinav Arun Sonkar, Donzília Sousa Silva, Ernesto Sparrelid, Parthi Srinivasan, Malin Sternby Eilard, Oliver Strobel, Urban Stupan, Miguel Angel Suarez-Munoz, Manisekar Subramaniam, Teiichi Sugiura, Robert Sutcliffe, Hilko Swank, Lillian Taylor, Prabin Bikram Thapa, Catherine The, Asara Thepbunchonchai, Caman Thieu, Navneet Tiwari, Guido Torzilli, Chutwichai Tovikkai, Blaz Trotovsek, Savvas Tsaramanidis, Georgios Tsoulfas, Katsuhiko Uesaka, Garzali Umar, Lucio Urbani, Michail Vailas, Ronald van Dam, Peter van de Boezem, Stijn van Laarhoven, Tomas Vanagas, Mike Van Dooren, Manon Viennet, Luca Vigano, Aarathi Vijayashanker, Celia Villodre, Toshifumi Wakai, Aklile Workneh, Li Xu, Masakazu Yamamoto, Zhiying Yang, Robert Young, Marko Zivanovic

**Affiliations:** a Department of HPB Surgery, Cambridge University Hospitals NHS Foundation Trust, Hills Road, Cambridge CB2 0QQ, United Kingdom; b Cambridge Clinical Trials Unit – Cancer Theme, Cambridge University Hospitals NHS Foundation Trust, Hills Road, Cambridge CB2 0QQ, United Kingdom; c Department of Statistics, Athens University of Economics and Business, Athens, Greece; d Department of Oncology, Cambridge University Hospitals NHS Foundation Trust, Hills Road, Cambridge CB2 0QQ, United Kingdom; e Department of Digestive Surgery, Hepato-Pancreato-Biliary Surgery Unit, Surgery Service, Gallbladder Consortium Chile, Sotero del Rio Hospital and Clinica Alemana, Santiago, Chile; f Department of Surgery, University of Buenos Aires, Hospital Dr Cosme Argerich, Buenos Aires, Argentina; g Department of Surgery, Emory University School of Medicine, Atlanta, GA 30322 USA; h Department of HPB Surgery, Hospital Sotero del Rio, Av. Concha y Toro 3459, Puente Alto, Región Metropolitana, Chile; i Department of Surgery, Lund University, Skane University Hospital, Lund, Sweden; j Department of HPB Surgery, Miguel Servet University Hospital, Zaragoza, Spain; k Department of Pathology, Koç University Hospital, Istanbul 34010, Turkey

**Keywords:** Gallbladder cancer, Liver resection, Surgical outcomes, Cholangiocarcinoma

## Abstract

**Background:**

Gallbladder cancer (GBC) is rare but aggressive. The extent of surgical intervention for different GBC stages is non-uniform, ranging from cholecystectomy alone to extended resections including major hepatectomy, resection of adjacent organs and routine extrahepatic bile duct resection (EBDR). Robust evidence here is lacking, however, and survival benefit poorly defined. This study assesses factors associated with recurrence-free survival (RFS), overall survival (OS) and morbidity and mortality following GBC surgery in high income countries (HIC) and low and middle income countries (LMIC).

**Methods:**

The multicentre, retrospective Operative Management of Gallbladder Cancer (OMEGA) cohort study included all patients who underwent GBC resection across 133 centres between 1st January 2010 and 31st December 2020. Regression analyses assessed factors associated with OS, RFS and morbidity.

**Findings:**

On multivariable analysis of all 3676 patients, wedge resection and segment IVb/V resection failed to improve RFS (HR 1.04 [0.84–1.29], p = 0.711 and HR 1.18 [0.95–1.46], p = 0.13 respectively) or OS (HR 0.96 [0.79–1.17], p = 0.67 and HR 1.48 [1.16–1.88], p = 0.49 respectively), while major hepatectomy was associated with worse RFS (HR 1.33 [1.02–1.74], p = 0.037) and OS (HR 1.26 [1.03–1.53], p = 0.022). Furthermore, EBDR (OR 2.86 [2.3–3.52], p < 0.0010), resection of additional organs (OR 2.22 [1.62–3.02], p < 0.0010) and major hepatectomy (OR 3.81 [2.55–5.73], p < 0.0010) were all associated with increased morbidity and mortality. Compared to LMIC, patients in HIC were associated with poorer RFS (HR 1.18 [1.02–1.37], p = 0.031) but not OS (HR 1.05 [0.91–1.22], p = 0.48). Adjuvant and neoadjuvant treatments were infrequently used.

**Interpretation:**

In this large, multicentre analysis of GBC surgical outcomes, liver resection was not conclusively associated with improved survival, and extended resections were associated with greater morbidity and mortality without oncological benefit. Aggressive upfront resections do not benefit higher stage GBC, and international collaborations are needed to develop evidence-based neoadjuvant and adjuvant treatment strategies to minimise surgical morbidity and prioritise prognostic benefit.

**Funding:**

Cambridge Hepatopancreatobiliary Department Research Fund.

**Research in context**

**Evidence before this study**

Gallbladder cancer (GBC) is a rare malignancy worldwide for which there are no robust data to guide curative strategies. The benefits of operative intervention on survival from GBC, particularly more extensive resections, are uncertain. We searched PubMed from 1st January 1990 to 1st January 2022 for articles in English using the search term “gallbladder cancer”. There are no randomised trials or large prospective cohort studies assessing surgical outcomes from GBC. Retrospective studies using individual national cancer statistics lacked detail in important confounding factors such as comorbidities and types of operative intervention. Most studies featured small patient numbers or centred on high income countries (HICs), and little information was available on outcomes from low and middle income countries (LMICs). Moreover, conclusions from some studies were not consistent with current guidelines for operative intervention for GBC.

**Added value of this study**

This is the largest cohort study (n = 3676) examining the outcomes from surgical intervention for GBC, spanning 133 centres from 41 countries in HICs and LMICs and including regions with high and low incidence of GBC. Extensive resections (i.e., major hepatectomy, routine bile duct excision or resection of other organs) were associated with an excess risk of significant complications or death for 1 in 4 patients undergoing these procedures compared to cholecystectomy alone, with no associated improvement in overall survival. Higher tumour stage, nodal involvement and positive margins were associated with poorer prognosis. Liver resection showed no association with a survival benefit for the majority of GBC tumour stages and neoadjuvant and adjuvant treatments were poorly utilised across the whole cohort.

**Implications of all the available evidence**

Aggressive surgical approaches for GBC continue to be practiced globally against international consensus guidelines and without overall survival benefit. This contemporary study corroborates findings from previous small studies suggesting cholecystectomy alone is sufficient for most early stage tumours. Higher tumour stages and those with nodal involvement are unlikely to benefit from surgery given the likelihood of systemic disease, and should be considered for neoadjuvant treatment modalities. Current outcomes from surgical management of GBC in LMICs are broadly comparable to HICs. Multicentre studies via international collaborative research networks are needed to improve neoadjuvant and adjuvant management strategies and develop globally relevant guidelines to improve outcomes from GBC.

## Introduction

Gallbladder cancer (GBC) is an aggressive malignancy, and the most common cancer of the biliary tract.^1^ There are substantial variations in incidence worldwide, but overall it is rare.^1^^,^^2^ There have thus been no randomised controlled trials (RCTs) or large prospective studies on GBC to guide surgical intervention. Most studies to date have been relatively small, and the few larger registry studies, such as those utilising the SEER database, contain non-standardized data and insufficient detail to avoid bias from confounding factors.^3^^,^^4^ Global health studies have demonstrated disparities between cancer outcomes in low and middle income countries (LMICs) and high income countries (HICs), but there is little data on this for GBC.^5^

The lack of robust data in this field has resulted in heterogenous management strategies for different stages of GBC. One particular area of controversy is the need for liver resection in addition to cholecystectomy for pre-operatively diagnosed GBC. Recent meta-analyses appear to contradict current guidelines on the need for liver resection for T1b disease, and debate continues regarding any improvement in recurrence-free survival (RFS) with liver resection in T2 disease.^3^^,^^4^^,^^6^ There is particular discussion around the optimal extent of liver resection, which can range from removing a rim of liver to more major surgery resecting approximately two-thirds of liver volume.

While neoadjuvant treatment is increasingly used in oesophageal and rectal cancers to reduce the extent of surgical intervention and risk of recurrence, it has been employed much less frequently for GBC, most probably due to the paucity of sound RCT evidence in this field.^7^, ^8^, ^9^ Indeed, studies indicate that many hepatopancreatobiliary (HPB) surgeons globally are more likely to adopt aggressive surgical options for locally advanced GBC than follow published consensus guidelines recommending systemic treatment, including a recent survey wherein over 30% stated they would routinely perform major hepatectomy or extrahepatic bile duct resection (EBDR) for higher stage tumours.^10^, ^11^, ^12^, ^13^, ^14^ The morbidity of these operations for patients is considerable, as well as the additional cost to healthcare systems, and any additional survival benefit is poorly-defined.^13^

The aim of this study was to obtain data on the operative management of GBC on a global scale, leveraging large sample sizes from multiple centres across HICs and LMICs. The main objective of the study was to assess factors associated with RFS and overall survival (OS) for GBC following surgery with curative intent. A secondary objective was to assess factors associated with perioperative morbidity and mortality.

## Methods

### Recruitment, data collection, and inclusion criteria

Collaborating centres were recruited by invitation disseminated via emails to all members of the three international HPB associations (the European-African HPB Association, the Americas HPB Association, and the Asia–Pacific HPB Association).

#### Inclusion and exclusion criteria

Patients who had undergone surgery for pre-operatively diagnosed or incidental GBC (identified following cholecystectomy for benign disease) were included in this study. Histological staging was classified according to the 8th American Joint Committee on Cancer (AJCC) classification (2018) for GBC (Supplementary Table S1a and b).^15^ Exclusion criteria were high-grade dysplasia with no invasive disease, metastatic disease at the time of surgery, or macroscopic tumour remaining at the end of resection (R2 resections).

#### Demographic and pathologic data collection

Clinical parameters, operative details, pathological findings as well as follow-up and survival data were obtained from institutional databases. Comorbidities were incorporated in the Charlson Comorbidity Index (CCI). Country income levels were classified according to the four 2021 World Bank categories of high income countries (HICs) and upper-middle, lower-middle or low income countries (the latter three groups amalgamated to a single category of low or middle income countries, LMICs).^16^ Incidence of GBC was determined using Globocan 2020 data, using the top quartile (≥1.1 GBC cases per 100,000 population) as the threshold for “high” incidence on multivariable analyses.^2^

#### Operative details

Extent of surgery was defined as cholecystectomy only (liver resection not performed), wedge resection (taking a margin of liver at the gallbladder bed), resection of liver segments IVb and V, or major hepatectomy (right, extended left or extended right hemihepatectomy). Data was also obtained on EBDR, resection of additional organs and surgical approach (open, robotic or laparoscopic, the latter two classified as “minimally-invasive”). Complications occurring either within 30 or 90 days of surgery were classified using the Clavien-Dindo scale (Supplementary Table S2).^17^

#### Follow-up and survival

OS was defined as the interval between the date of surgery for GBC (for patients who underwent further surgery for incidental GBC, the date of the second operation was used) and the date of death, obtained from hospital or government records at the time of study closure. RFS was defined as the time interval between the date of surgery and either the date of first identification of recurrence on imaging or histology or the date of most recent imaging excluding recurrence.

### Ethical approval

Institutional and ethical approval was obtained from the Research and Development Office at Cambridge University Hospitals NHS Foundation Trust (the lead site) and the United Kingdom Research Ethics Committee (IRAS ID 285918). Informed consent from patients was deemed unnecessary by the ethics committee for this retrospective study. Other participating centres obtained further institutional and national approvals as needed. This study was conducted and reported in compliance with the STROBE guidelines for cohort studies.^18^

### Statistical analysis

Median follow up was calculated from the Kaplan–Meier estimate and associated 95% confidence intervals. Putative prognostic parameters for RFS, OS, morbidity and mortality covering demographic, oncological and treatment-related factors were identified from the literature. Association between these parameters and RFS and OS was assessed using Cox proportional hazards multivariable regression models, with all relevant assumptions met. Multivariable logistic regression was performed to assess associations with morbidity and mortality, using the complications within the first 30 days after surgery with a Clavien–Dindo score of IIIA and above as a binary endpoint, termed “30-day severe morbidity and mortality”. The parameter “income” showed collinearity with “regional GBC incidence”, while “minimally-invasive approach” showed collinearity with T, N and R stage, and thus those combinations of parameters were not used as variables in the same multivariable model. Model selection was performed using Akaike Information Criterion (AIC)-based stepwise methods.

Sensitivity analyses were performed replicating multivariable analyses using individual T-stages to assess applicability of findings from the whole cohort analysis. Subgroup univariable analysis was performed to assess effects of extent of liver resection on RFS and OS by T-stage, and effects of chemotherapy on RFS and OS for individual AJCC stages. Chi-squared tests and Fisher's exact test were used to assess for significant differences between categorical variables on subgroup analyses.

All statistical analyses were conducted in R statistical software.

### Role of the funding source

The funder had no role in study design, data collection, data analysis, data interpretation or writing of the report.

## Results

**Fig. 1:**
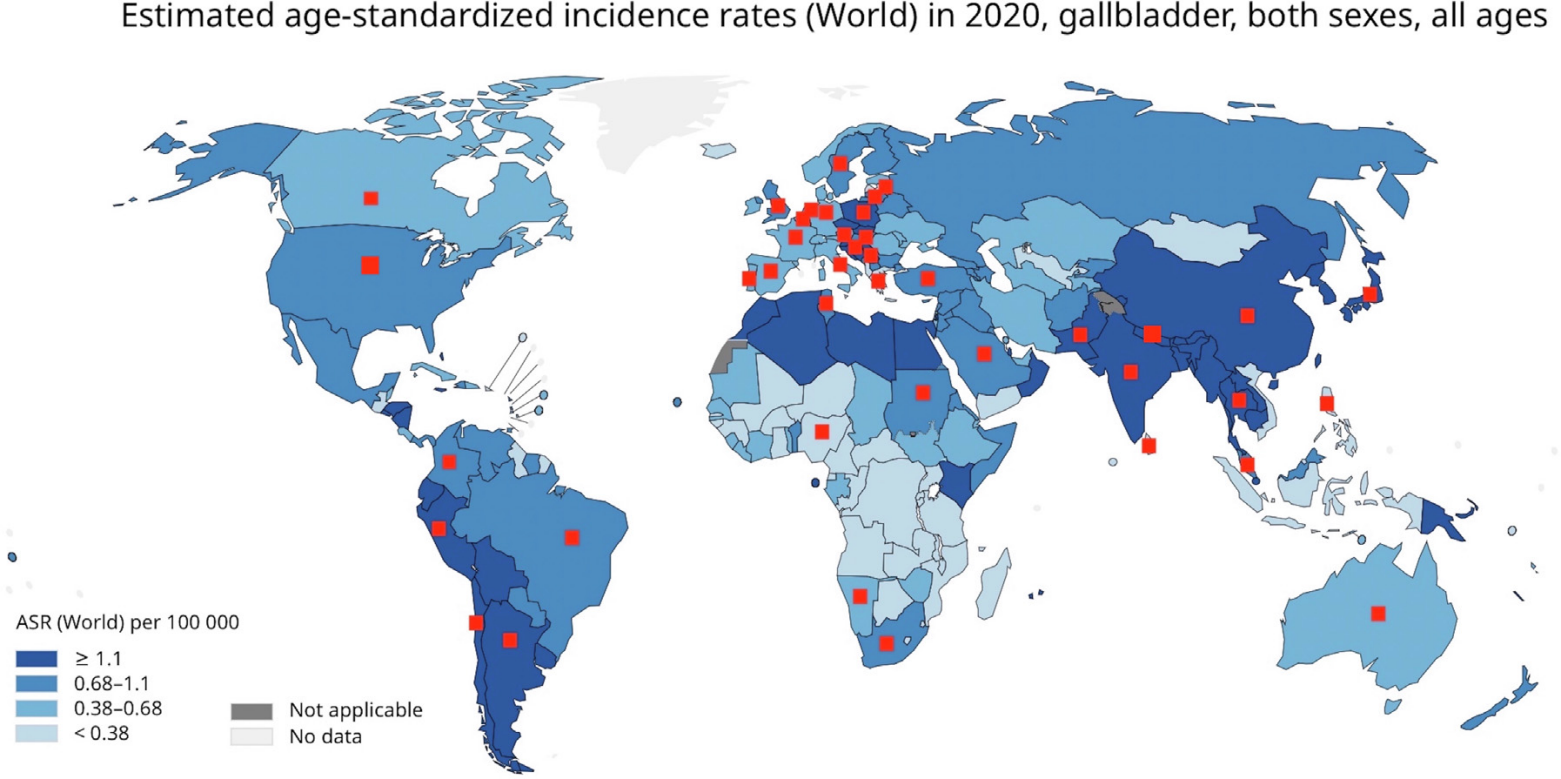
**Distribution of countries participating in the OMEGA study (marked by red squares) superimposed on estimated global age-standardized incidence rates (ASR) of gallbladder cancer per 100,000 individuals.** Adapted with permission from the International Agency for Research on Cancer's GLOBOCAN database, ref. 2, IARC.

**Fig. 2:**
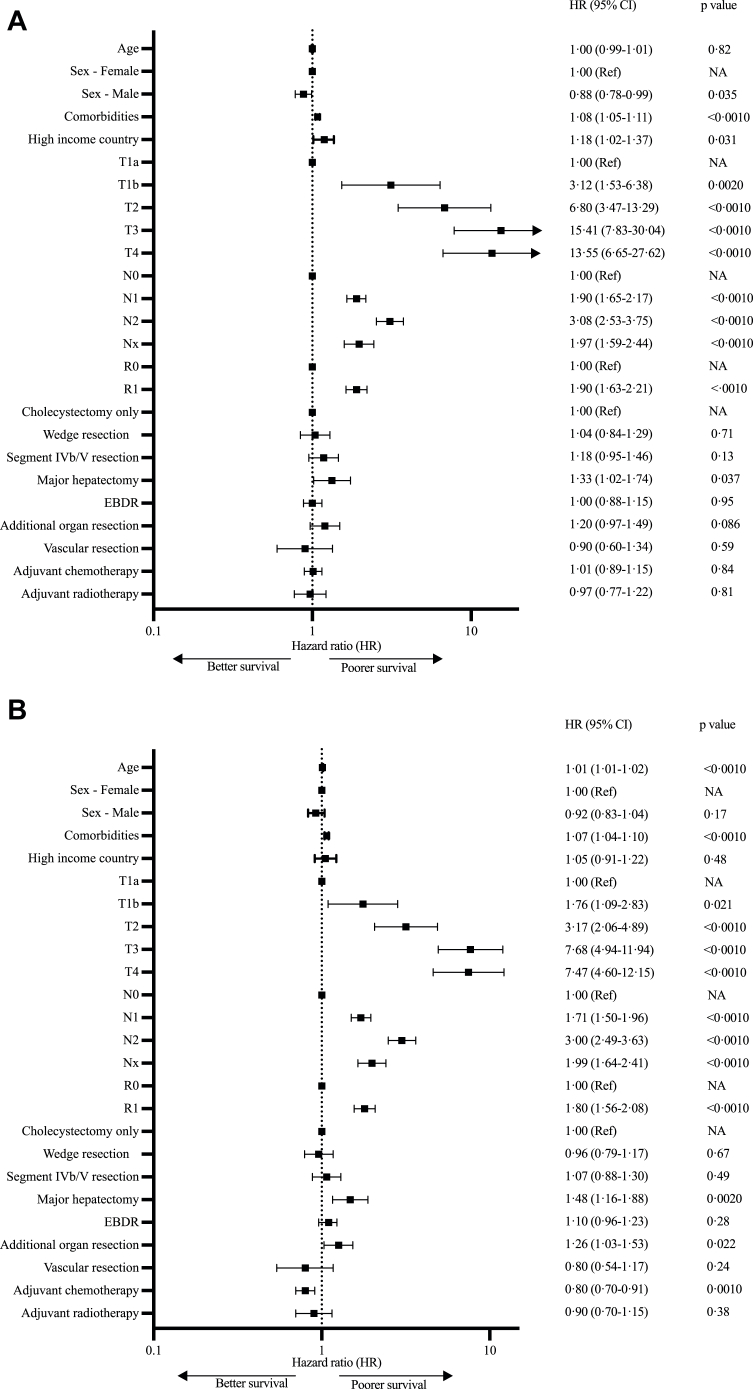
**Forest plots and multivariable Cox proportional hazard regression analysis of factors influencing RFS (A) and OS (B).** EBDR – extrahepatic bile duct resection, CI – confidence intervals.

A total of 4138 patients were identified who had undergone surgery for GBC at 133 participating centres in 41 countries, between 1st January 2010 and 31st December 2020 (Fig. 1). Three hundred and eighty-four patients with macroscopic tumour remaining after surgery or metastatic disease and 78 patients with only high-grade dysplasia were excluded, leaving a final total of 3676 patients for analysis (n = 2787 from HIC, Table 1, Supplementary Tables S3 and S4). Median age at time of surgery was 66 (interquartile range 58–74) years. OS and RFS were available for 3626 and 2993 patients respectively. Median follow-up duration was 45.3 (interquartile range 24.1–80.5) months. Median OS and RFS for the full cohort were 51.2 (49.3–52.8) months and 35.2 (34.3–36.9) months, respectively. HICs had a lower incidence of GBC than LMICs (Supplementary Table S3), and treatment in HICs was associated with worse RFS compared to LMICs (HR 1.18 [95% CI 1.02–1.37], p = 0.031), but not OS (HR 1.05 [0.91–1.22], p = 0.48, Fig. 2). Fewer patients undergoing surgery in LMICs had significant comorbidities (CCI scores of 7 and above) compared to those in HICs (4.6%, n = 42, vs 17.6%, n = 492 respectively, p < 0.0010, Supplementary Table S3). When income was substituted with national GBC incidence within the multivariable model, treatment in countries with a high GBC incidence, vs low, was associated with better RFS and OS (HR 0.86 [0.76–0.97], p = 0.015 and HR 0.85 [0.75–0.96], p = 0.0070 respectively).

**Table 1:** Demographic data, with percentages in brackets.

Variable	Number of patients (%)
Sex - Male	1252 (34.1%)
**Age**^a^	
<40	108 (2.9%)
40–59	936 (25.4%)
60–79	2269 (61.7%)
≥80	360 (9.8%)
**Income level**	
High	2787 (75.8%)
Non-high	889 (24.2)
Upper middle	363 (9.9%)
Lower middle	516 (14.0%)
Low	10 (0.3%)
**Incidence of GBC**	
High (≥1.1 cases per 100,000 population)	1456 (39.6%)
Low (<1.1 cases per 100,000 population)	2220 (60.4%)
**Charlson Comorbidity Index**	
0–3	1500 (40.8%)
4–6	1642 (44.7%)
7–10	287 (7.8%)
>10	25 (0.7%)
Unknown	222 (6.0%)
**pT category**^b^	
pT1a	187 (5.1%)
pT1b	403 (11.0%)
pT2	1700 (46.2%)
pT3	1186 (32.3%)
pT4	200 (5.4%)
**pN category**^b^	
N0	1874 (51.0%)
N1	1081 (29.4%)
N2	272 (7.4%)
Nx	449 (12.2%)
**pR category**^c^	
R0	3187 (86.7%)
R1	472 (12.8%)
Unknown	17 (0.5%)

Data is presented as absolute number (percentage).

Abbreviation: GBC – gallbladder cancer.

a Age at time of cholecystectomy.

b AJCC definitions presented in Supplementary Table S1a.

c R1 indicates positive margins on resection.

### RFS and OS are primarily associated with T-stage, N-stage and margin status

Higher T stage, nodal involvement and positive margins were associated with greatest risk of impaired OS and RFS on multivariable analysis (Fig. 2, Supplementary Fig. S1a–c). Nx status (no nodes resected) conferred a hazard ratio on RFS similar to that of N1 disease (HR 1.97 [1.59–2.44], p < 0.0010 and HR 1.90 [1.65–2.17], p < 0.0010), and occurred less frequently in LMICs (7.9%, n = 70 vs 13.6%, n = 379, p < 0.0010). T2b disease (tumours on hepatic side of the gallbladder, n = 276) was associated with poorer survival compared to T2a (tumours located on the peritoneal side, n = 239) on subgroup analysis, with 3 year RFS of 54.5% ± 4.3% vs 67.7% ± 4.3%, p = 0.0030, and 3 year OS 68.0% ± 3.6% vs 77.7% ± 3.4%, p = 0.044, however numbers of patients undergoing specific interventions in these two groups were too small for further comparative analyses (Supplementary Table S5).

**Fig. 3:**
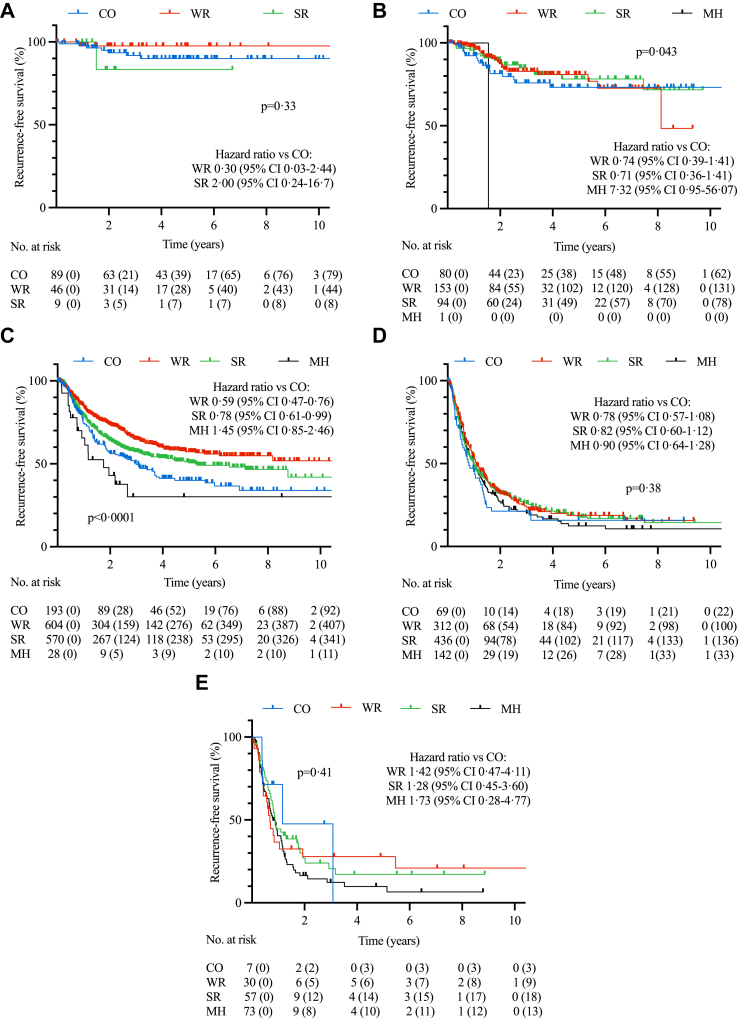
**Recurrence-free survival (RFS) curves for T1a (A), T1b (B), T2 (C), T3 (D) and T4 (E) stages of GBC according to extent of surgery performed.** CO – cholecystectomy only, WR – wedge resection, SR – segment IVb/V resection, MH – major hepatectomy. Censored numbers in brackets. p-value of Kaplan–Meier curve calculated by log-rank test and hazard estimates by univariable Cox proportional hazards model compared to “cholecystectomy only”.

Addition of liver resection (wedge resection, segment IVb/V resection or major hepatectomy) was not associated with improved RFS or OS compared to cholecystectomy alone on multivariable analysis of the entire cohort, or multivariable analysis of individual T-stages in sensitivity analyses (Fig. 2 and Supplementary Fig. S2a–e). Multivariable analysis of patients with N0 disease only similarly failed to identify an associated improvement in RFS for liver resection over cholecystectomy alone for any T-stage (data not shown). On subgroup univariable analysis, wedge resections and segment IVb/V resections were associated with improved RFS compared to cholecystectomy alone for T2 disease only (HR 0.59 [0.47–0.76] and HR 0.78 [0.61–0.99], p < 0.0001, Fig. 3).

EBDR was not associated with improved RFS or OS across the whole cohort (HR 1.00 [0.88–1.15], p = 0.95 and HR 1.10 [0.96–1.89], p = 0.28 respectively, Fig. 2) or for any individual T-stage (Supplementary Fig. S2a–e). Resection of other organs including pancreas, stomach or colon was not associated with better RFS on multivariable analysis (HR 1.20 [0.97–1.49], p = 0.086) but was associated with worse OS (HR 1.26 [1.03–1.53], p = 0.022, Fig. 2).

### Extended resections confer the greatest risk of morbidity and mortality

**Fig. 4:**
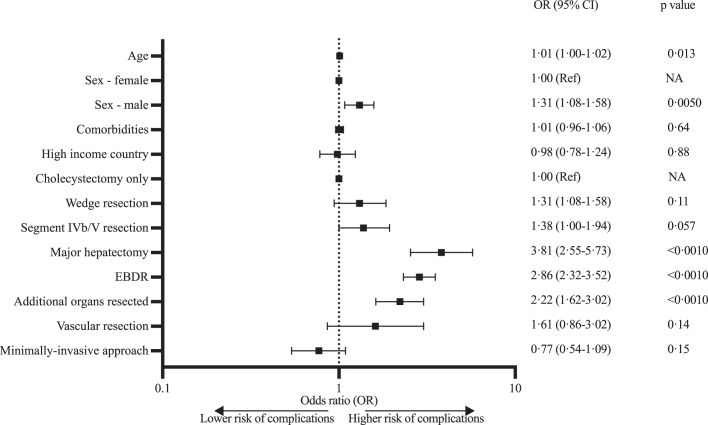
**Forest plot and multivariable logistic regression analysis of factors influencing 30-day severe morbidity and mortality (Clavien-Dindo Grade IIIA and above).** EBDR – extrahepatic bile duct resection, CI – confidence intervals, OR – odds ratio.

All-cause 30-day and 90-day mortality was 1.9% (70 patients) and 4.1% (149 patients) respectively. The most common specific causes of 90-day mortality were liver failure (n = 29, 19.5%) especially following major hepatectomy (n = 25 patients), and haemorrhage (n = 18, 12.1%). Major hepatectomy and resection of additional organs were each independently associated with increased 30-day severe morbidity and mortality on multivariable logistic regression analysis (OR 3.81 [2.55–5.73], p < 0.0010, and OR 2.22 [1.62–3.02], p < 0.0010 respectively, Fig. 4). EBDR was associated with a more than two-fold increased risk of bile leak by (18.9% vs 7.6%, p < 0.0010), as well as increased 30-day severe morbidity and mortality (OR 2.86 [2.32–3.52], p < 0.0010, Fig. 4). No difference was noted between HICs and LMICs in the rate of 30-day severe morbidity and mortality on multivariable analysis (Fig. 4).

### Low utilisation of neoadjuvant or adjuvant treatment modalities

Only 2.4% of patients (n = 90) received neoadjuvant chemotherapy, while none received neoadjuvant radiotherapy (Supplementary Table S4). Adjuvant chemotherapy (received by 1212 patients, 33.0%) was not associated with improved RFS on multivariable analysis of the whole cohort (HR 1.01 [0.89–1.15], p = 0.85), but was associated with improved OS (HR 0.80 [0.70–0.91], p = 0.0010, Fig. 2, regimes shown in Supplementary Table S6). On subgroup analysis by AJCC stages, adjuvant chemotherapy was associated with higher RFS for stage IVB disease (patients with N2 disease, p < 0.0010) and higher OS for patients with disease stages IIIB and above (p < 0.05, Supplementary Tables S1b and S7). Adjuvant radiotherapy (received by 158 patients, 4.3%, Supplementary Table S4) was not associated with improved OS (HR 0.90 [0.70–1.15], p = 0.38) or RFS (HR 0.97 [0.77–1.22], p = 0.81) for the overall cohort on multivariable analysis, or for any individual T stage on subgroup analysis (p > 0.050, Fig. 2 and Supplementary Fig. S2a–e). Adjuvant chemotherapy was received by almost twice the proportion of LMIC patients compared to HIC patients (50.5%, n = 449, vs 27.4%, n = 763, p < 0.0010) but a smaller proportion of LMIC patients received radiotherapy (3.7%, n = 33, vs 4.5%, n = 125, p = 0.020, Supplementary Table S4).

## Discussion

In this largest, contemporary study of surgical resection for GBC in multiple tertiary international institutions, we demonstrate that higher tumour stages, nodal involvement and positive margins were associated with poorer survival, and liver resection was not conclusively associated with improved survival over cholecystectomy alone. Major hepatectomy, EBDR or additional organ resection were all associated with significant increases in morbidity and mortality with no associated improvement in survival, and adjuvant and neoadjuvant treatment options were infrequently utilised.

While T1a tumours are considered treatable by cholecystectomy alone, current guidelines recommend liver resection for stage T1b, hitherto considered aggressive tumours based on SEER registry data reporting low survival rates.^6^^,^^11^^,^^12^ This may, however, be due to undersampling of the gallbladder in some centres, resulting in underdiagnosis of true cases of T2 disease. Indeed more recent studies including a meta-analysis have shown better survival rates for T1b tumours and good outcomes associated with cholecystectomy alone.^4^^,^^19^ Our data support these latter findings showing no benefit associated with liver resection for T1b disease. Variation in survival data for early stage tumours may also be related to substantial subjectivity and geographic differences in the pathological classification of non-invasive vs minimally invasive (T1) carcinomas, and international collaborations are underway to establish standardized descriptive criteria to better characterize the behaviour of early GBC.^20^

There is more consistency between the literature and current guidelines for T2 tumours, suggesting a potential benefit for liver resection, but any associated survival benefit is only shown on univariable analysis and lost on multivariable analysis in our study and others.^11^^,^^12^^,^^21^ This may partly be explained by confounding effects of positive margins, nodal status or comorbidities on univariable analysis. A more pertinent issue may be that the hazard benefit associated with liver resection in T2 disease in our multivariable analysis was small, and would require a study population of approximately 3367 patients with T2 disease to identify a significant difference in RFS – unlikely to be easily achieved given the rare nature of GBC. Tumour location within the gallbladder (hepatic vs peritoneal side) has also been postulated to affect survival, but although T2b disease was associated with worse RFS in our study compared to T2a, this was not corroborated in a recent meta-analysis.^1^^,^^3^ A recent development that may also influence the benefit associated with liver resection is the identification of prognostic correlation between sub-stages of tumour depth (which can range from several microns deep to those several centimeters in depth for T2 disease).^15^^,^^22^ More studies are clearly warranted to better risk stratify subgroups likely to benefit from liver resection, and until then we would advocate that liver resection should continue to be performed for T2 disease with judicious patient selection.

Liver resection in T3 and T4 tumours was not associated with improved OS or RFS. While it could be argued that the T4 cohort had insufficient patient numbers to detect an associated survival benefit, this was not a limitation for the much larger T3 cohort. The greater likelihood of nodal and margin involvement and micrometastatic spread at higher disease stages may therefore obviate any benefit from liver resection at higher tumour stages, corroborating similar findings from other studies.^13^^,^^23^ These data should not be interpreted to suggest that cholecystectomy alone should be considered sufficient treatment for these higher stages of disease, but rather that these patients may benefit more from neoadjuvant chemotherapy rather than upfront surgery.

More extensive liver resection, specifically major hepatectomy, was associated with the highest risk of morbidity and mortality in this study, predominantly due to liver insufficiency, consistent with previous data on this procedure for other tumour types.^24^ It was not possible to draw any conclusions regarding the benefit of segment IVb/V resections over wedge resections in this study, as the exact extent of resection performed in each of these two procedures in practice may be imprecise. Our findings correlating extent of resection with increased morbidity would suggest that, where appropriate, the least extensive liver resection necessary to achieve clear margins should be performed. EBDR was associated with increased complication rates and poorer RFS, corroborating a recent meta-analysis, and possibly due to post-operative morbidity jeopardising adjuvant treatment.^14^ For the majority of patients EBDR is therefore either unnecessary due to early stage disease, or insufficient in higher stages to clear lymphatic and perineural tumour involvement along the biliary tract, and hence these procedures, as for other extended resections such as major hepatectomy, should be very selectively adopted in centres with appropriate expertise.^11^^,^^12^ Notwithstanding the above controversies regarding extent of liver resection, there is far greater consensus regarding the importance of lymph node assessment for staging and adjuvant treatment. Nodal status was strongly associated with RFS and OS, leading us to concur with recommendations that all patients undergoing surgery for T1b GBC and above should have adequate regional lymphadenectomy, and those not undergoing surgery should have endoscopic or radiologic nodal staging to guide further treatment.^11^^,^^12^

Downstaging or neoadjuvant therapy is now frequently used in other gastrointestinal malignancies such as rectal and oesophagogastric cancer to improve tumour-free margins and OS.^7^^,^^8^ This has been recommended for GBC for higher disease stages, but remains very infrequently used in standard practice globally and often biased towards academic centres.^11^^,^^12^^,^^25^ Recent studies have shown an association between neoadjuvant chemotherapy and improved RFS following subsequent surgery, and results of ongoing RCTs are eagerly awaited.^25^, ^26^, ^27^, ^28^ Adjuvant chemotherapy in this study was associated with improved OS for T3 and T4 disease but not for RFS, however this may have been related to small patient numbers and multiple treatment regimens. Both adjuvant chemotherapy and radiotherapy were infrequently used in our study, possibly due to availability or post-operative fitness, but also influenced by relatively little evidence for adjuvant chemotherapy until the per-protocol analysis of the BILCAP phase III study demonstrating a benefit for capecitabine vs observation alone for biliary tract cancers (of which 18% had GBC).^9^^,^^11^^,^^12^ No RCTs exist for adjuvant radiotherapy, and current recommendations for radiotherapy for patients with R1 margins are based on retrospective studies and the single-arm SWOG S0809 trial.^29^ Future trials focusing specifically on GBC alone, exploring targeted therapies in the context of comprehensive (and even potentially region-specific) genomic profiling, or incorporating newer strategies such as immune checkpoint inhibitors showing promising results in advanced biliary tract cancers, may identify better roles for these approaches in addition to surgical intervention.^30^

We noted some interesting differences between GBC management in patients from HICs and LMICs in this study, for example the association of poorer RFS with HICs, possibly related to surveillance bias due to insufficient or inconsistent access to early surveillance imaging in LMICs.^5^ Our study covered many centres across a range of healthcare systems with varying models of healthcare funding and service delivery however, and therefore many other environmental and socioeconomic factors may play a contributory role. Further studies are clearly needed to explore cancer outcomes from LMICs in greater detail, nevertheless this study has now established a GBC research network, incorporating both HICs and LMICs, likely to prove beneficial in initiating future collaborative trials and ensuring that any new treatment guidelines are applicable on an international scale.

The key strength of this study resides in the large patient cohort from multiple international centres, incorporating sufficient detail, such as comorbidities, often missing from large national cancer registries. Nevertheless there are some unavoidable limitations. Some countries remain under-represented, and the deliberate inclusion of only HPB specialist cancer centres may have missed some patients (particularly T1a tumours) who underwent cholecystectomy at non-specialist hospitals and were never referred. The reason for cholecystectomy was not always available, and therefore comparisons between outcomes from incidental vs per primum GBC could not be made. Very limited data could be obtained regarding demographic risk factors such as smoking, alcohol consumption and body mass index and thus these factors could not be analysed in this study. It was not possible to adjust for inevitable variation in factors such as healthcare systems and resources across cities or case volume, resources and technical expertise across centres. Information on recurrence was not available on patients followed up elsewhere, and management practices were heterogenous across centres, both with regards to operative extent as well as utilisation of neoadjuvant or adjuvant treatment.

This study is the first to assess operative outcomes from GBC across such a large cross-section of countries and hospitals, and reflects current practice with real-world challenges and limitations. Given the lack of RCTs or large-scale prospective studies for this rare cancer, our data currently provides the best available evidence on the utility of various resection options in the management of GBC. Our findings show that liver resection was not conclusively associated with improved survival, and extended resections were associated with increased morbidity and mortality without clearly associated oncological benefit. We would advocate moving away from aggressive resections for GBC, particularly for higher stages of disease, and greater emphasis on neoadjuvant strategies and collaborative international multicentre trials. This approach would mark a paradigm shift in the global management of GBC, away from surgical morbidity and towards multimodality treatment strategies emphasising maximal prognostic benefit.

## Contributors

A.B., A.S. and V.A. conceived the study. A.B., A.J., B.B., J.L., S.M., B.A., A.S. and V.A. formed the expert panel. A.B., H.V.M.S., S.T., J.L.M., B.A., A.S., A.J., R.K.P., S.J.F.H., S.S.L., V.K., P.G., E.L.H. and A.Br. coordinated the study conduct and data collection. P.B., N.D. and A.B. did the data analyses and verified the data. A.B., A.J., B.B., B.A., J.L., S.M., A.Br., X.d.A., A.S. and V.A. drafted the manuscript with assistance from all co-authors. All authors critically assessed the study design, enrolled patients in the study, edited the manuscript, and approved the final manuscript. The corresponding author had final responsibility for the decision to submit for publication.

The OMEGA study investigators are co-authors in this study and the list of investigators in this group is provided separately.

## Data sharing statement

Data ownership for the data compiled in the OMEGA study remains the property of individual OMEGA collaborating units, and data sharing with third parties will therefore not be possible without permission from respective individual contributors.

## Declaration of interests

SM has the following interests: 1) Principal investigator of a clinical trial on intrahepatic cholangiocarcinoma funded by BMS/Celgene (grant funding provided to institution, no personal compensation) 2) Advisory board member at Astra Zeneca on management of hepatocellular carcinoma and 3) Scientific Medical Advisory Board member Cholangiocarcinoma Foundation.

None of the authors have any conflicts of interests to declare.

## Appendix A Supplementary data

Supplementary data

OMEGA Study Investigators List

## References

[1] Roa J.C., García P., Kapoor V.K., Maithel S.K., Javle M., Koshiol J. (2022). Gallbladder cancer. Nat Rev Dis Primers.

[2] Ferlay J., Ervik M., Lam F. (2020). https://gco.iarc.fr/today.

[3] Alrawashdeh W., Kamarajah S.K., Gujjuri R.R. (2022). Systematic review and meta-analysis of survival outcomes in T2a and T2b gallbladder cancers. HPB (Oxford).

[4] Lee H., Kwon W., Han Y., Kim J.R., Kim S.-W., Jang J.-Y. (2018). Optimal extent of surgery for early gallbladder cancer with regard to long-term survival: a meta-analysis. J Hepatobiliary Pancreat Sci.

[5] Meara J.G., Leather A.J.M., Hagander L. (2015). Global Surgery 2030: evidence and solutions for achieving health, welfare, and economic development. Lancet.

[6] Vogel A., Bridgewater J., Edeline J. (2023). Biliary tract cancer: ESMO clinical practice guideline for diagnosis, treatment and follow-up. Ann Oncol.

[7] Lagergren J., Smyth E., Cunningham D., Lagergren P. (2017). Oesophageal cancer. Lancet.

[8] Zhang X., Ma S., Guo Y., Luo Y., Li L. (2022). Total neoadjuvant therapy versus standard therapy in locally advanced rectal cancer: a systematic review and meta-analysis of 15 trials. PLoS One.

[9] Bridgewater J., Fletcher P., Palmer D.H. (2022). Long-term outcomes and exploratory analyses of the randomized phase III BILCAP study. J Clin Oncol.

[10] Balakrishnan A., Jah A., Lesurtel M. (2022). Heterogeneity of management practices surrounding operable gallbladder cancer – results of the OMEGA-S international HPB surgical survey. HPB (Oxford).

[11] Aloia T.A., Járufe N., Javle M. (2015). Gallbladder cancer: expert consensus statement. HPB (Oxford).

[12] Benson A.B., D'Angelica M.I., Abrams T.A. (2014). Hepatobiliary cancers, version 2.2014. J Natl Compr Canc Netw.

[13] Fancellu A., Sanna V., Deiana G. (2021). Current role of hepatopancreatoduodenectomy for the management of gallbladder cancer and extrahepatic cholangiocarcinoma: a systematic review. World J Gastrointest Oncol.

[14] Lv T.-R., Liu F., Hu H.-J. (2022). The role of extra-hepatic bile duct resection in the surgical management of gallbladder carcinoma. A first meta-analysis. Eur J Surg Oncol.

[15] Amin M. (2017).

[16] World Bank country and lending groups. https://datahelpdesk.worldbank.org/knowledgebase/articles/906519-world-bank-country-and-lending-groups?report=reader.

[17] Dindo D., Demartines N., Clavien P.-A. (2004). Classification of surgical complications: a new proposal with evaluation in a cohort of 6336 patients and results of a survey. Ann Surg.

[18] von Elm E., Altman D.G., Egger M. (2007). The Strengthening the Reporting of Observational Studies in Epidemiology (STROBE) statement: guidelines for reporting observational studies. Lancet.

[19] Kim H.S., Park J.W., Kim H. (2018). Optimal surgical treatment in patients with T1b gallbladder cancer: an international multicenter study. J Hepatobiliary Pancreat Sci.

[20] Pehlivanoglu B., Akkas G., Memis B. (2023). Reappraisal of T1b gallbladder cancer (GBC): clinicopathologic analysis of 473 in situ and invasive GBCs and critical review of the literature highlights its rarity, and that it has a very good prognosis. Virchows Arch.

[21] Kwon W., Kim H., Han Y. (2020). Role of tumour location and surgical extent on prognosis in T2 gallbladder cancer: an international multicentre study. Br J Surg.

[22] Memis B, Roa JC, Muraki T, et al. Not all T2 gallbladder carcinomas (GBC) are equal: proposal for sub-staging of T2 GBC with significant prognostic value (Abstract).

[23] Tharmalingam S., Flemming J., Richardson H., Hurlbut D., Cleary S., Nanji S. (2022). Surgical practice patterns and outcomes in T2 and T3 gallbladder cancer: a population-based study. Can J Surg.

[24] Baumgartner R., Gilg S., Björnsson B. (2022). Impact of post-hepatectomy liver failure on morbidity and short- and long-term survival after major hepatectomy. BJS Open.

[25] Ozer M., Goksu S.Y., Sanford N.N. (2022). A propensity score analysis of chemotherapy use in patients with resectable gallbladder cancer. JAMA Netw Open.

[26] Goetze T.O., Bechstein W.O., Bankstahl U.S. (2020). Neoadjuvant chemotherapy with gemcitabine plus cisplatin followed by radical liver resection versus immediate radical liver resection alone with or without adjuvant chemotherapy in incidentally detected gallbladder carcinoma after simple cholecystectomy or in front of radical resection of BTC (ICC/ECC) - a phase III study of the German registry of incidental gallbladder carcinoma platform (GR)- the AIO/CALGP/ACO- GAIN-trial. BMC Cancer.

[27] The OPT-IN study, Clinical Trials.gov Identifier:NCT04559139. NCT04559139.

[28] Chaudhari V.A., Ostwal V., Patkar S. (2018). Outcome of neoadjuvant chemotherapy in ‘locally advanced/borderline resectable’ gallbladder cancer: the need to define indications. HPB (Oxford).

[29] Ben-Josef E., Guthrie K.A., El-Khoueiry A.B. (2015). SWOG S0809: a phase II intergroup trial of adjuvant capecitabine and gemcitabine followed by radiotherapy and concurrent capecitabine in extrahepatic cholangiocarcinoma and gallbladder carcinoma. J Clin Oncol.

[30] Oh D.-Y., Ruth He A., Qin S. (2022). Durvalumab plus gemcitabine and cisplatin in advanced biliary tract cancer. NEJM Evid.

