# Supplementary material for: Surgical outcomes of gallbladder cancer: the OMEGA retrospective, multicentre, international cohort study
Source: eClinicalMedicine. 2023 Apr 13;59:101951. doi: 10.1016/j.eclinm.2023.101951 (PMC10130604; doi:10.1016/j.eclinm.2023.101951)
Supplement: Supplementary data [file mmc1.docx]

**Supplementary tables and figures:**

Supplementary table 1a: 8^th^ American Joint Committee on Cancer (AJCC) pathological classification

| **T category** | **T criteria** |
| --- | --- |
| Tx | Primary tumour cannot be assessed |
| T0 | No evidence of primary tumour |
| Tis | Carcinoma in situ |
| T1a | Tumour invades the lamina propria |
| T1b | Tumour invades the muscular layer |
| T2 | Tumour invades the perimuscular connective tissue on the peritoneal side, without involvement of the serosa (visceral peritoneum)  Or tumour invades the perimuscular connective tissue on the hepatic side with no extension into the liver |
| T2a | Tumour invades the perimuscular connective tissue on the peritoneal side, without involvement of the serosa (visceral peritoneum) |
| T2b | Tumour invades the perimuscular connective tissue on the hepatic side with no extension into the liver |
| T3 | Tumour perforates the serosa (visceral peritoneum) and /or directly invades the liver and/or one other adjacent organ or structure, such as the stomach, duodenum, colon, pancreas, omentum or extrahepatic bile ducts |
| T4 | Tumour invades the main portal vein or hepatic artery or invades two or more extrahepatic organs or structures |
|  |  |
| **N category** | **N criteria** |
| Nx | Regional lymph nodes cannot be assessed |
| N0 | No regional lymph node metastasis |
| N1 | Metastases to one to three regional lymph nodes |
| N2 | Metastases to four or more regional lymph nodes |
|  |  |
| **M category** | **M criteria** |
| M0 | No distant metastasis |
| M1 | Distant metastasis |

Supplementary table 1b: 8^th^ American Joint Committee on Cancer (AJCC) prognostic stage groups

| Stage | T | N | M |
| --- | --- | --- | --- |
| 0 | Tis | N0 | M0 |
| I | T1 | N0 | M0 |
| IIA | T2a | N0 | M0 |
| IIB | T2b | N0 | M0 |
| IIIA | T3 | N0 | M0 |
| IIIB | T1-3 | N1 | M0 |
| IVA | T4 | N0-1 | M0 |
| IVB | Any T | N2 | M0 |
| IVB | Any M | Any N | M1 |

Supplementary Table 2: Clavien-Dindo Classification of post-operative complications

| Grade I | Any deviation from the normal postoperative course without the need for pharmacological treatment or surgical, endoscopic and radiological interventions  Allowed therapeutic regimens are: drugs as antiemetics, antipyretics, analgesics, diuretics, electrolytes and physiotherapy. This grade also includes wound infections opened at the bedside. |
| --- | --- |
| Grade II | Requiring pharmacological treatment with drugs other than such allowed for Grade I complications. Blood transfusions and total parenteral nutrition are also included |
| Grade III  Grade IIIA  Grade IIIB | Requiring surgical, endoscopic or radiological intervention  Intervention not under general anaesthesia  Intervention not under general anaesthesia |
| Grade IV  Grade IVA  Grade IVB | Life-threatening complication (including central nervous system complications) requiring intensive care unit management  Single organ dysfunction (including dialysis)  Multiorgan dysfunction |
| Grade V | Death of a patient |

Adapted from reference 17 - Dindo D, Demartines N, Clavien P-A. Classification of surgical complications: a new proposal with evaluation in a cohort of 6336 patients and results of a survey. *Ann Surg* 2004; **240**: 205–13.

Supplementary table 3: Demographic data by high income (HIC) versus low and middle income (LMIC) countries

| **Variable** | **HIC (n=2787)** | **LMIC (n=889)** | **p value** |
| --- | --- | --- | --- |
| Sex - Male | 954 (34·2%) | 298 (33·5%) | 0·70 |
| **Incidence of GBC** |  |  |  |
| High (>1·1 cases per 100,000 population) | 688 (24·7%) | 768 (86·4%) | <0·0010 |
| Low (< 1·1 cases per 100,000 population) | 2099 (75·3%) | 121 (13·6%) |  |
| **Charlson Comorbidity Index (CCI)** |  |  |  |
| 0-3 | 968 (34·7%) | 532 (59·8%) | <0·0010 |
| 4-6 | 1327 (47·6%) | 315 (35·4%) |  |
| 7-10 | 251 (9·0%) | 36 (4·0%) |  |
| >10 | 23 (0·8%) | 2 (0·2%) |  |
| Unknown | 218 (7·8%) | 4 (0·4%) |  |
| **pT category** |  |  |  |
| pT1a | 157 (5·6%) | 30 (3·4%) | 0·0020 |
| pT1b | 292 (10·5%) | 111 (12·5%) |  |
| pT2 | 1318 (47·3%) | 382 (43·0%) |  |
| pT3 | 878 (31·5%) | 308 (34·6%) |  |
| pT4 | 142 (5·1%) | 58 (6·5%) |  |
| **pN category** |  |  |  |
| N0 | 1431 (51·3%) | 443 (49·8%) | <0·0010 |
| N1 | 798 (28·6%) | 283 (31·8%) |  |
| N2 | 179 (6·4%) | 93 (10·5%) |  |
| Nx | 379 (13·6%) | 70 (7·9%) |  |
| **pR category** |  |  |  |
| R0 | 2382 (85·5%) | 805 (90·6%) | <0·0010 |
| R1 | 392 (14·1%) | 80 (9·0%) |  |
| Unknown | 13 (0·5%) | 4 (0·4%) |  |

Data is presented as absolute number (percentage). Abbreviations: GBC; gallbladder cancer. HIC; high income country. LMIC; low or middle income country. P values denote comparisons between HIC and LMIC, calculated with the χ^2^ test.

| **Variable** | **Overall cohort**  **(n=3676)** | **HIC**  **(n=2787)** | **LMIC**  **(n=889)** | **p value** |
| --- | --- | --- | --- | --- |
| **Extent of liver resection** |  |  |  |  |
| Not performed (cholecystectomy only) | 557 (15·1%) | 491 (17·6%) | 66 (7·4%) | <0·0010 |
| Wedge resection | 1407 (38·3%) | 988 (35·5%) | 419 (47·1%) |  |
| Segment IVb/V resection | 1397 (38·0%) | 1062 (38·1%) | 335 (37·7%) |  |
| Major hepatectomy | 315 (8·6%) | 246 (8·8%) | 69 (7·7%) |  |
| Right hemihepatectomy | 293 (8·0%) | 231 (8·3%) | 64 (7·2%) |  |
| Extended left hemihepatectomy | 10 (0·3%) | 6 (0·2%) | 4 (0·4%) |  |
| Extended right hemihepatectomy | 10 (0·3%) | 9 (0·3%) | 1 (0·1%) |  |
| **Additional organs resected** |  |  |  |  |
| Hemicolectomy / segmental colectomy | 94 (2·6%) | 60 (2·2%) | 34 (3·8%) | 0·018 |
| Distal gastrectomy/ localised duodenectomy | 74 (2·0%) | 51 (1·8%) | 23 (2·6%) |  |
| Pancreatectomy/ pancreaticoduodenectomy | 106 (2·9%) | 74 (2·7%) | 32 (3·6%) |  |
| None | 3432 (93·3%) | 2619 (94·0%) | 813 (91·4%) |  |
| **Vascular resection** |  |  |  |  |
| Arterial (right hepatic artery, common hepatic artery) | 14 (0·4%) | 14 (0·5%) | 0 | 0·060 |
| Portal venous | 36 (1·0%) | 30 (1·1%) | 6 (0·7%) |  |
| Not performed | 3626 (98·6%) | 2743 (98·4%) | 883 (99·3%) |  |
| **Operative approach** |  |  |  |  |
| Open | 3162 (86·0%) | 2362 (84·8%) | 800 (90·0%) | 0·0010 |
| Laparoscopic | 465 (12·6%) | 379 (13·6%) | 86 (9·7%) |  |
| Robotic | 36 (1·0%) | 33 (1·2%) | 3 (0·3%) |  |
| Unknown | 13 (0·4%) | 13 (0·5%) | 0 |  |
| **Clavien-Dindo complication grade** |  |  |  |  |
| None | 1870 (50·9%) | 1500 (53·8%) | 370 (41·6%) | <0·0010 |
| I | 461 (12·5%) | 295 (10·6%) | 166 (18·7%) |  |
| II | 594 (16·2%) | 408 (14·6%) | 186 (20·9%) |  |
| IIIA | 364 (9·9%) | 283 (10·2%) | 81 (9·1%) |  |
| IIIB | 137 (3·7%) | 103 (3·7%) | 34 (3·8%) |  |
| IVA | 84 (2·9%) | 68 (2·4%) | 16 (1·8%) |  |
| IVB | 37 (1·0%) | 33 (1·2%) | 4 (0·4%) |  |
| V | 70 (1·9%) | 46 (1·7%) | 24 (2·7%) |  |
| **Neoadjuvant chemotherapy** |  |  |  |  |
| Received neoadjuvant chemotherapy | 90 (2·4%) | 75 (2·7%) | 15 (1·7%) | 0·092 |
| No neoadjuvant chemotherapy | 3586 (97·6%) | 2712 (97·3%) | 874 (98·3%) |  |
| **Neoadjuvant radiotherapy** |  |  |  |  |
| Received neoadjuvant radiotherapy | 0 | 0 | 0 | NA |
| No neoadjuvant radiotherapy | 3676 (100%) | 2787 (100%) | 889 (100%) |  |
| **Adjuvant chemotherapy** |  |  |  |  |
| Received chemotherapy | 1212 (33·0%) | 763 (27·4%) | 449 (50·5%) | <0·0010 |
| No chemotherapy | 2253 (61·3%) | 1877 (67·3%) | 376 (42·3%) |  |
| Unknown | 211 (5·7%) | 147 (5·3%) | 64 (7·2%) |  |
| **Adjuvant radiotherapy** |  |  |  |  |
| Received radiotherapy | 158 (4·3%) | 125 (4·5%) | 33 (3·7%) | 0·020 |
| No radiotherapy | 3311 (90·1%) | 2521 (90·5%) | 790 (88·9%) |  |
| Unknown | 207 (5·6%) | 141 (5·1%) | 66 (7·4%) |  |

Supplementary table 4: Operative and post-operative parameters for high and non-high income countries

Data is presented as absolute number (percentage). Abbreviations: GBC; gallbladder cancer. HIC; high income country. LMIC; low or middle income country. P values denote comparisons between HIC and LMIC, calculated with the χ^2^ test.

**Supplementary figure 1: Recurrence-free survival according to T stage (A), N stage (B) and R status (C).** . Censored numbers in brackets. P-value of Kaplan-Meier curve calculated by log-rank test and hazard estimates by univariable Cox proportional hazards model compared to stated baselines.


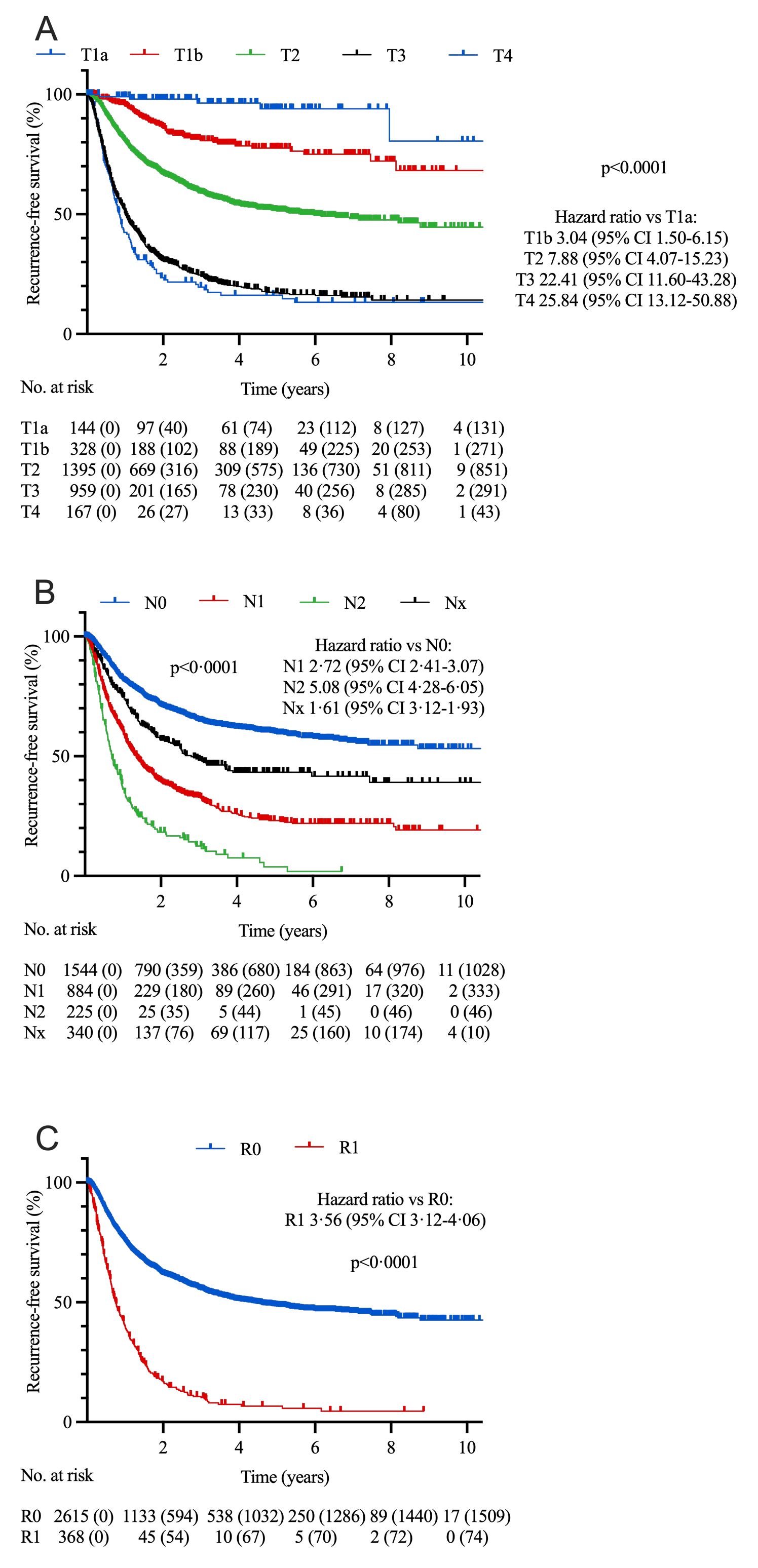


Supplementary Figure 2a: Forest plot and multivariable Cox proportional hazard regression analysis of factors influencing recurrence-free survival (RFS, A) and overall survival (OS, B) for T1a disease. EBDR – extrahepatic bile duct resection, CI – confidence intervals, HR – hazard ratio


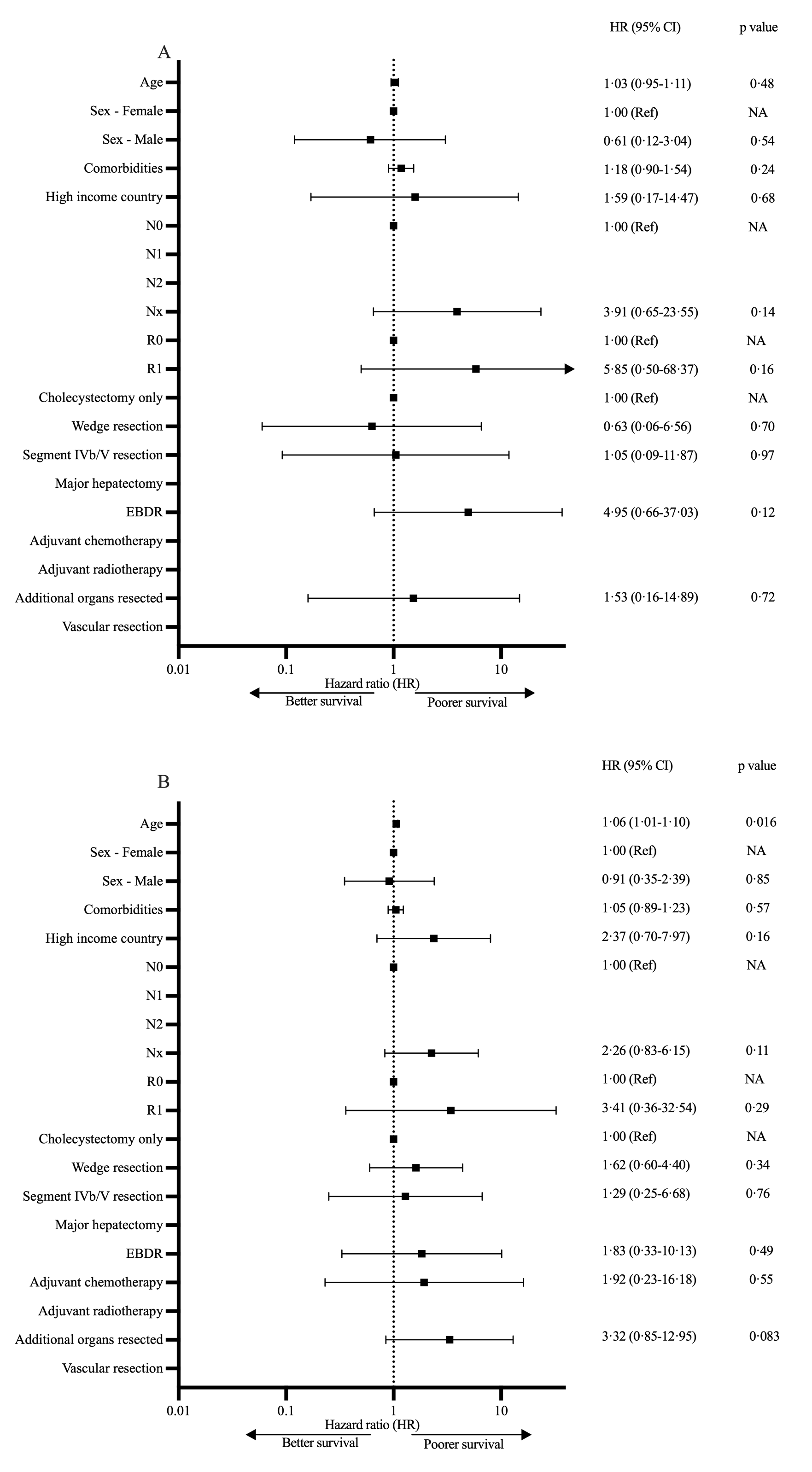


Supplementary Figure 2b: Forest plot and multivariable Cox proportional hazard regression analysis of factors influencing recurrence-free survival (RFS, A) and overall survival (OS, B) for T1b disease. EBDR – extrahepatic bile duct resection, CI – confidence intervals, HR – hazard ratio


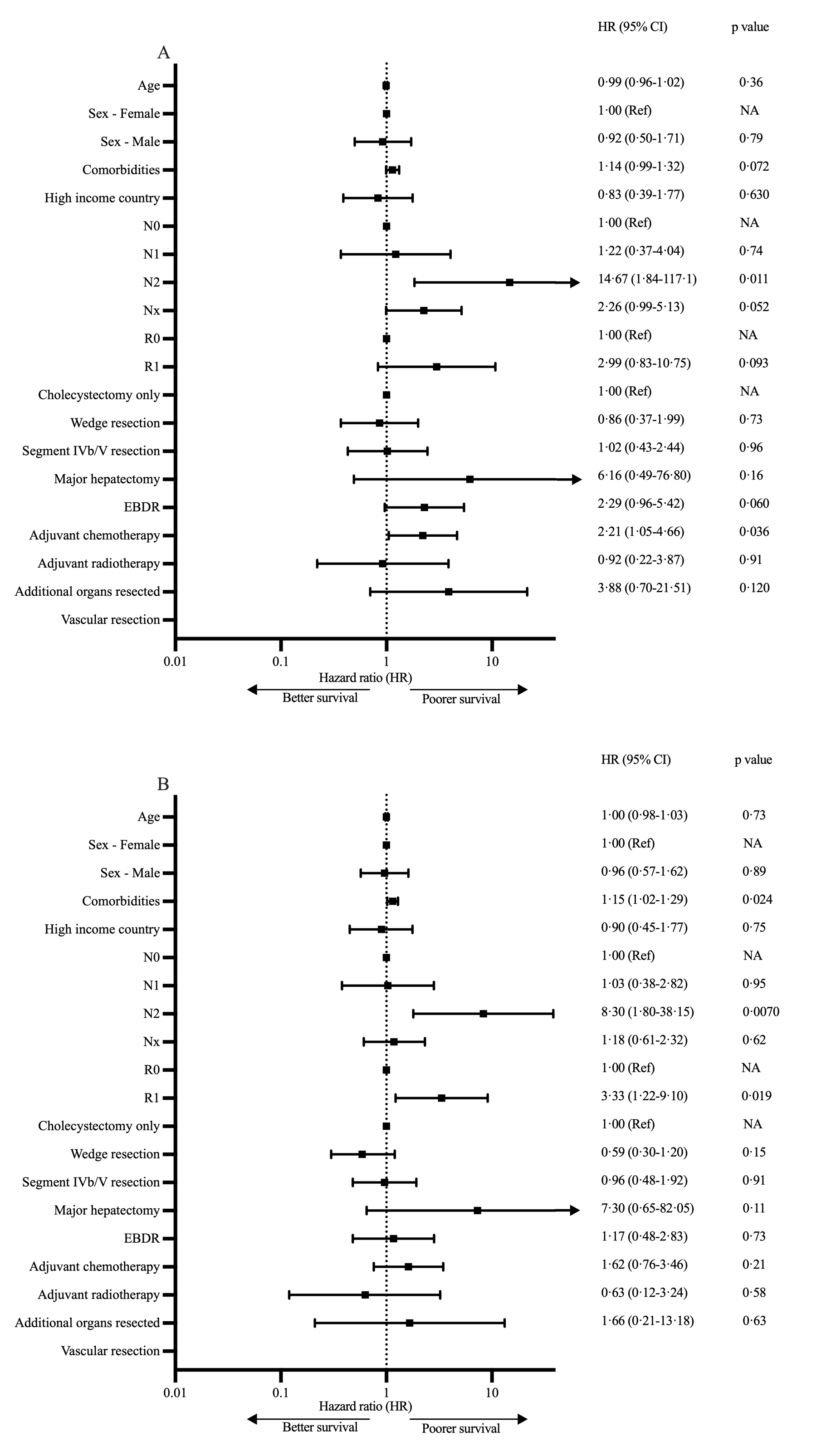


Supplementary Figure 2c: Forest plot and multivariable Cox proportional hazard regression analysis of factors influencing recurrence-free survival (RFS, A) and overall survival (OS, B) for T2 disease. EBDR – extrahepatic bile duct resection, CI – confidence intervals, HR – hazard ratio


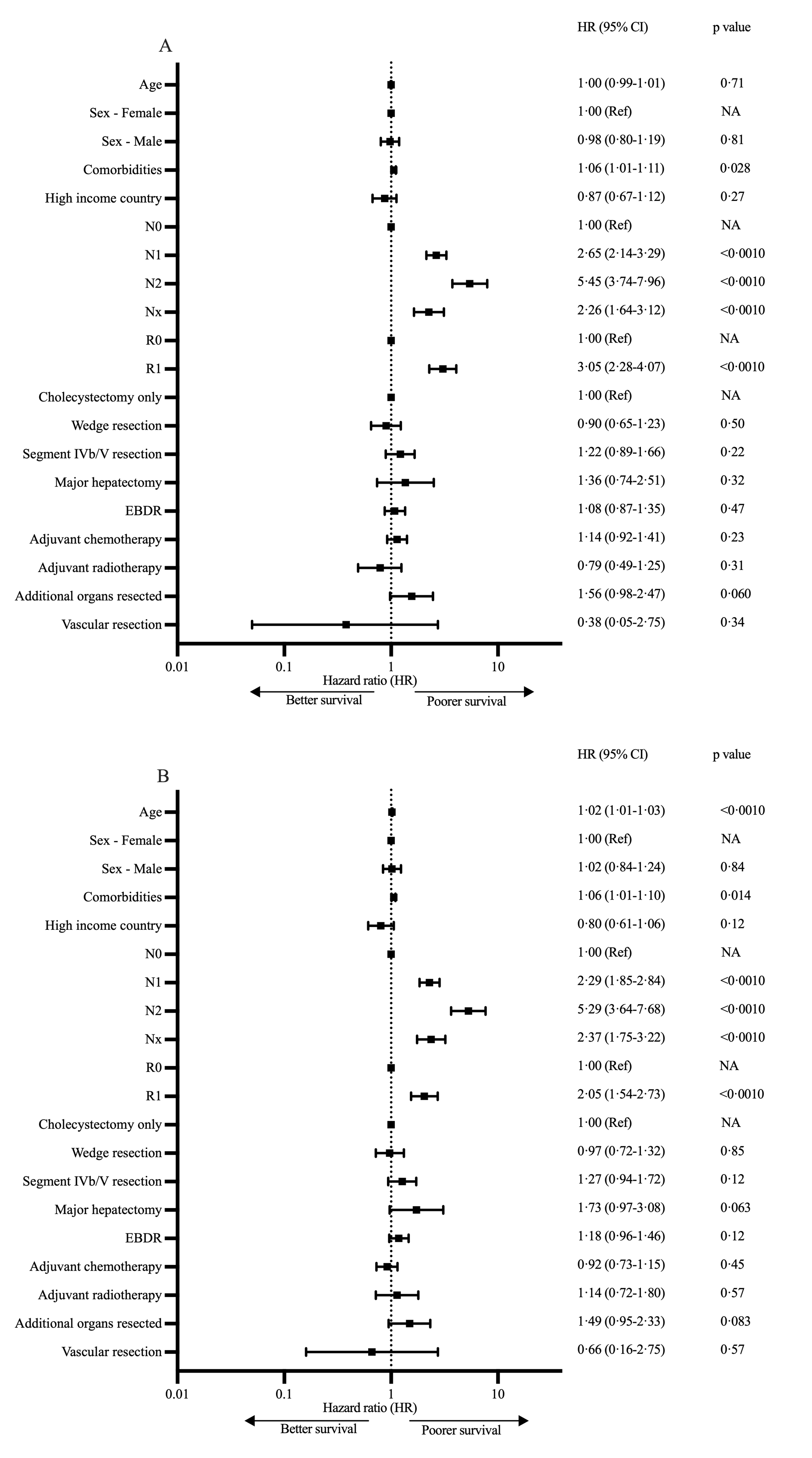


Supplementary Figure 2d: Forest plot and multivariable Cox proportional hazard regression analysis of factors influencing recurrence-free survival (RFS, A) and overall survival (OS, B) for T3 disease. EBDR – extrahepatic bile duct resection, CI – confidence intervals, HR – hazard ratio


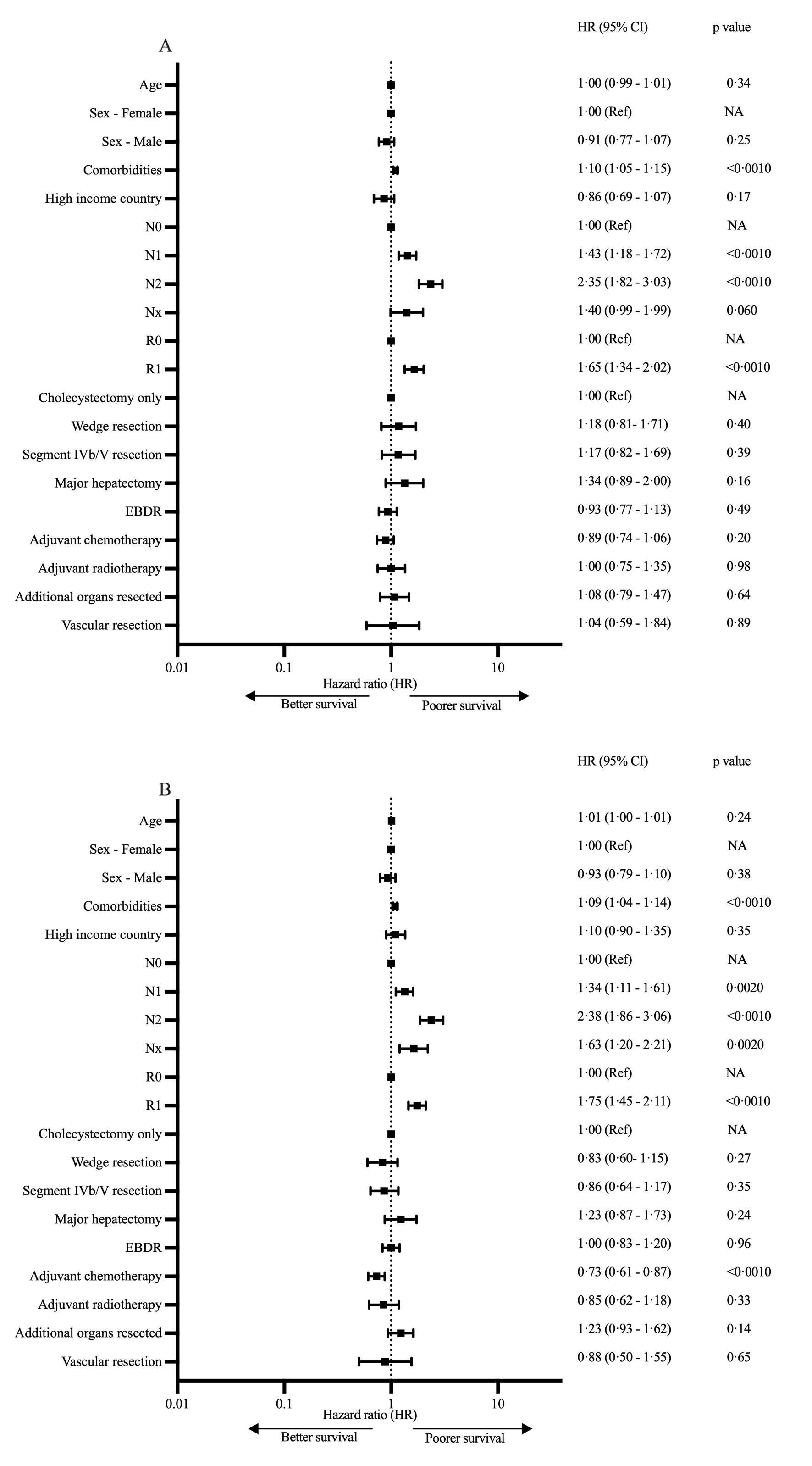


Supplementary Figure 2e: Forest plot and multivariable linear regression analysis of factors influencing recurrence-free survival (RFS, A) and overall survival (OS, B) for T4 disease. EBDR – extrahepatic bile duct resection, CI – confidence intervals, HR – hazard ratio


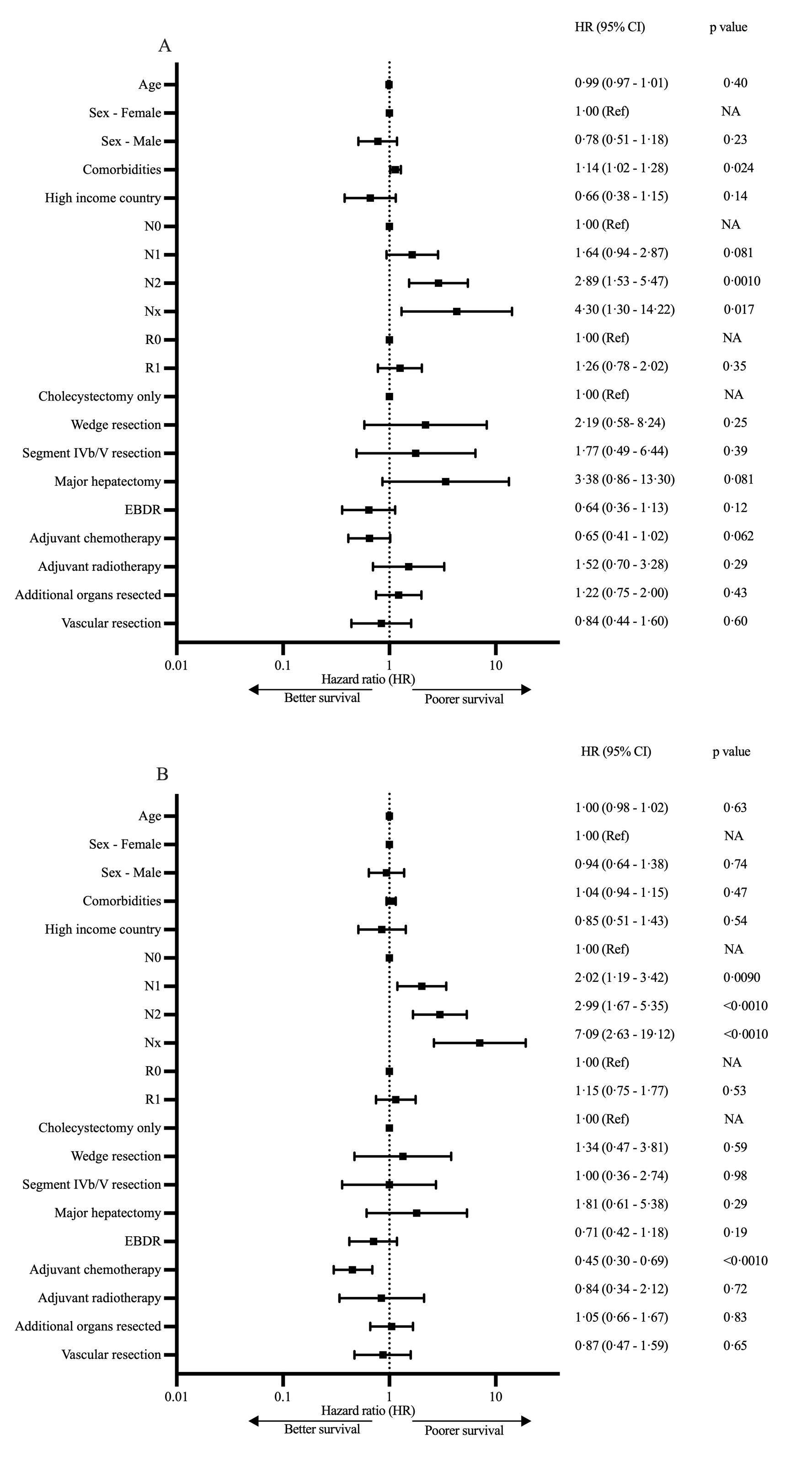


Supplementary Table 5:

| T2 substage | 3-year RFS | Standard error | p value | 3-year OS | Standard error | p value |
| --- | --- | --- | --- | --- | --- | --- |
| T2a (n=239) | 67·7% | 4·3% | p=0·003 | 77·7% | 3·4% | p=0·044 |
| T2b (n=276) | 54·5% | 4·3% |  | 68·0% | 3·6% |  |

Supplementary table 6: Adjuvant chemotherapy regimens given for GBC in high income countries and low or middle income countries.

| **Adjuvant chemotherapy regimen** | **Overall (n=3676)** | **HIC (n=2787)** | **LMIC (n=889)** |
| --- | --- | --- | --- |
| Monotherapy regimens | | | |
| Capecitabine | 362 (9·8%) | 284 (10·2%) | 78 (8·8%) |
| 5FU | 37 (1·0%) | 23 (0·8%) | 14 (1·6%) |
| Gemcitabine | 138 (3·8%) | 111 (4·0%) | 27 (3·0%) |
| S1 (tegafur/ gimeracil/ oteracil) | 77 (2·1%) | 70 (2·5%) | 7 (0·8%) |
|  | | | |
| Combination therapy regimens | | | |
| Capecitabine + oxaliplatin | 7 (0·2%) | 6 (0·2%) | 1 (0·1%) |
| Gemcitabine + Cisplatin | 430 (11·7%) | 148 (5·3%) | 282 (31·7%) |
| Cisplatin/ Oxaliplatin | 8 (0·2%) | 6 (0·2%) | 2 (0·2%) |
| FOLFOX or FOLFIRINOX | 20 (0·5%) | 6 (0·2%) | 14 (1·6%) |
|  | | | |
| Regimen unknown | 133 (3·6%) | 109 (3·9%) | 24 (2·7%) |

Data is presented as absolute number (percentage). Abbreviations: GBC; gallbladder cancer. HIC; high income country. LMIC; low or middle income country.

Supplementary table 7: Univariate analysis comparing recurrence-free survival (RFS) and overall survival (OS) associated with adjuvant chemotherapy and five year RFS and OS percentages as comparative indicators of survival.

| AJCC Stage | Adjuvant chemotherapy administered? | Recurrence-free survival (RFS) | | | Overall survival (OS) | | |
| --- | --- | --- | --- | --- | --- | --- | --- |
|  |  | 5YS (%) | SE (%) | p value | 5YS (%) | SE (%) | p value |
| I | No (n=319) | 87·1 | 2·6 | 0·16 | 82·0 | 2·7 | 0·20 |
|  | Yes (n=67) | 84·6 | 5·4 |  | 68·8 | 12·5 |  |
| II | No (n=675) | 68·3 | 2·4 | 0·14 | 70·2 | 2·2 | 0·84 |
|  | Yes (255) | 64·3 | 4·5 |  | 69·1 | 4·4 |  |
| IIIA | No (n=237) | 25·7 | 3·9 | 0·95 | 32·1 | 3·6 | 0·12 |
|  | Yes (n=180) | 24·4 | 4·1 |  | 30·9 | 4·8 |  |
| IIIB | No (n=474) | 25·2 | 2·8 | 0·20 | 31·1 | 2·7 | <0·0010 |
|  | Yes (n=435) | 21·8 | 3 |  | 35·5 | 3·2 |  |
| IVA | No (n=75) | 19·6 | 6·2 | 0·49 | 13·4 | 5·1 | 0·0080 |
|  | Yes (n=61) | 22·2 | 6·2 |  | 29·7 | 7·2 |  |
| IVB | No (n=117) | 0 | 0 | <0·0010 | 3·8 | 2·3 | <0·0010 |
|  | Yes (n=272) | 6·4 | 3·8 |  | 20 | 5 |  |

AJCC – American Joint Committee on Cancer

Supplementary Table 8: Full list of participating countries in the OMEGA study

Argentina

Australia

Austria

China

Belgium

Brazil

Canada

Chile

Colombia

France

Germany

Greece

Hungary

India

Italy

Japan

Latvia

Lithuania

Malaysia

Namibia

Nepal

Netherlands

Nigeria

Pakistan

Peru

Philippines

Poland

Portugal

Saudi Arabia

Serbia

Slovenia

South Africa

Spain

Sri Lanka

Sudan

Sweden

Thailand

Tunisia

Turkey

United Kingdom

United States of America
