# Supplementary material for: Surgical outcomes of gallbladder cancer: the OMEGA retrospective, multicentre, international cohort study
Source: eClinicalMedicine. 2023 Apr 13;59:101951. doi: 10.1016/j.eclinm.2023.101951 (PMC10130604; doi:10.1016/j.eclinm.2023.101951)
Supplement: OMEGA Study Investigators List [file mmc2.docx]

OMEGA study investigators (collaborating co-authors):

| **First name** | **Last name** |
| --- | --- |
| Tomoyuki | Abe |
| Moh'd | Abu Hilal |
| Maria del Mar | Achalandabaso Boira |
| Mustapha | Adham |
| Mohamed | Adam |
| Maryam | Ahmad |
| Bilal | Al-Sarireh |
| Maite | Albiol |
| Nassir | Alhaboob |
| Adnan | Alseidi |
| Houssem | Ammar |
| Akshay | Anand |
| Bodil | Andersson |
| Pantelis | Antonakis |
| Veronica | Araya |
| Stanley W | Ashley |
| Georgi | Atanasov |
| Fabio | Ausania |
| Ricardo | Balestri |
| Abhirup | Banerjee |
| Sudeep  Simon | Banerjee  Banting |
| Giedrius | Barauskas |
| Fabian | Bartsch |
| Andrea | Belli |
| Simona | Beretta |
| Frederik | Berrevoet |
| Ramesh Singh  Gerardo | Bhandari  Blanco Fernandez |
| Louisa | Bolm |
| Mathieu | Bonal |
| Emre | Bozkurt |
| Andries E | Braat |
| Luke | Bradshaw |
| Konstantinos  Alejandro | Bramis  Branes |
| Lyle | Burdine |
| Matthew | Byrne |
| Maria | Caceres |
| Maria Jesus | Castro Santiago |
| Benjamin | Chan |
| Lynn | Chong |
| Ahmet | Çoker |
| Maria | Conde Rodriguez |
| Daniel | Croagh |
| Alyn | Crutchley |
| Carmen | Cutolo |
| Mathieu | D'Hondt |
| Daniel | D'Souza |
| Freek | Daams |
| Raffaele | Dalla Valle |
| José | Davide |
| Mario | de Bellis |
| Marieke | de Boer |
| Celine | de Meyere |
| Philip | de Reuver |
| Matthew | Dixon |
| Panagiotis | Dorovinis |
| Gabriela | Echeverría Bauer |
| Maria | Eduarda |
| Hasan | Eker |
| Joris | Erdmann |
| Mert | Erkan |
| Evangelos | Felekouras |
| Emanuele | Felli |
| Eduardo | Fernandes |
| Eduardo | Figueroa Rivera |
| Andras | Fulop |
| Daniel | Galun |
| Michael | Gerhards |
| Poya | Ghorbani |
| Fabio | Giannone |
| Luis | Gil |
| Emmanouil | Giorgakis |
| Mario | Giuffrida |
| Felice | Giuliante |
| Ioannis | Gkekas |
| Miguel | Gomez Bravo |
| Bas | Groot Koerkamp |
| Oscar | Guevara |
| Alfredo | Guglielmi |
| Aiste | Gulla |
| Rahul | Gupta |
| Amit | Gupta |
| Marta | Gutiérrez |
| Abu Bakar | Hafeez Bhatti |
| Jeroen | Hagendoorn |
| Zain | Hajee |
| Abdul Rahman | Hakeem |
| Hytham | Hamid |
| Sayed | Hassen |
| Stefan | Heinrich |
| Ryota | Higuchi |
| Daniel | Hoffman |
| David | Holroyd |
| Daniel | Hughes |
| Arpad | Ivanecz |
| Satheesh | Iype |
| Isabel | Jaen Torrejimeno |
| Shantanu | Joglekar |
| Robert | Jones |
| Klaus | Kaczirek |
| Harsh | Kanhere |
| Ambareen | Kausar |
| Zhanyi | Kee |
| Jessica | Keilson |
| Jorg | Kleef |
| Johannes | Klose |
| Brett | Knowles |
| Jun Kit | Koong |
| Nagappan | Kumar |
| Supreeth | Kunnuru |
| Paleswan Joshi | Lakhey |
| Andrea | Laurenzi |
| Yeong Sing | Lee |
| Felipe | Leon |
| Voon Meng | Leow |
| Jean-Baptiste | Lequeu |
| Mickael | Lesurtel |
| Elisabeth | Lo |
| Stefan | Löb |
| Elizabeth | Lockie |
| Peter | Lodge |
| Dolores | López Garnica |
| Victor | Lopez Lopez |
| Linda | Lundgren |
| Nikolaos | Machairas |
| Dhiresh | Maharjan |
| Deep | Malde |
| Guillaume | Martel |
| Julie | Martin |
| Michele | Mazzola |
| Arianeb | Mehrabi |
| Ricardo | Memeo |
| Flavio | Milana |
| George | Molina |
| Leah | Monette |
| Haluk | Morgul |
| Dimitrios | Moris |
| Antonios | Morsi-Yeroyannis |
| Nicholas | Mowbray |
| Francesk | Mulita |
| Edoardo Maria | Muttillo |
| Malith | Nandasena |
| Pueya Rashid | Nashidengo |
| Arash | Nickkholgh |
| Colin Byron | Noel |
| Masayuki | Ohtsuka |
| Arturs | Ozolins |
| Sanjay | Pandanaboyana |
| Nikolaos | Pararas |
| Alessandro | Parente |
| June | Peng |
| Arkaitz | Perfecto Valero |
| Julie | Perinel |
| Konstatinos | Perivoliotis |
| Teresa | Perra |
| Patrick | Pessaux |
| Natalie | Petruch |
| Gaetano | Piccolo |
| Laszlo | Piros |
| Alberto | Porcu |
| Viswakumar | Prabakaran |
| Raj | Prasad |
| Mikel | Prieto Calvo |
| Florian | Primavesi |
| Eva Maria | Pueyo Periz |
| Alberto | Quaglia |
| Jose M | Ramia Angel |
| Ashwin | Rammohan |
| Francesco | Razionale |
| Ricardo | Robles Campos |
| Manas | Roy |
| Sophie | Rozwadowski |
| Luis | Ruffolo |
| Natalia | Ruiz |
| Andrea | Ruzzenante |
| Lily | Saadat |
| Mohamed Amine | Said |
| Edoardo | Saladino |
| Gabriel | Saliba |
| Per | Sandstrom |
| Carlo Alberto | Schena |
| Anthony | Scholer |
| Christoph | Schwarz |
| Lorenzo | Serafini |
| Pablo E | Serrano |
| Deepak | Sharma |
| Aali | Sheen |
| Vishwanath | Siddagangaiah |
| Michael | Silva |
| Saurabh | Singh |
| Ajith | Siriwardena |
| Michal | Skalski |
| Mante | Smig |
| Faris | Soliman |
| Abhinav Arun  Donzília | Sonkar  Sousa Silva |
| Ernesto | Sparrelid |
| Parthi | Srinivasan |
| Malin | Sternby Eilard |
| Oliver | Strobel |
| Urban | Stupan |
| Miguel Angel | Suarez-Munoz |
| Manisekar | Subramaniam |
| Teiichi | Sugiura |
| Robert | Sutcliffe |
| Hilko | Swank |
| Lillian | Taylor |
| Prabin Bikram | Thapa |
| Catherine | The |
| Asara | Thepbunchonchai |
| Caman | Thieu |
| Navneet | Tiwari |
| Guido | Torzilli |
| Chutwichai | Tovikkai |
| Blaz | Trotovsek |
| Savvas | Tsaramanidis |
| Georgios | Tsoulfas |
| Katsuhiko | Uesaka |
| Garzali | Umar |
| Lucio | Urbani |
| Michail | Vailas |
| Ronald | van Dam |
| Peter | van de Boezem |
| Stijn | van Laarhoven |
| Tomas | Vanagas |
| Mike | Van Dooren |
| Manon | Viennet |
| Luca | Vigano |
| Aarathi | Vijayashanker |
| Celia | Villodre |
| Toshifumi | Wakai |
| Aklile | Workneh |
| Li | Xu |
| Masakazu | Yamamoto |
| Zhiying | Yang |
| Robert | Young |
| Marko | Zivanovic |
